# Supplementary material for: ZAK activation at the collided ribosome
Source: Nature. 2025 Nov 19;649(8098):1051–60. doi: 10.1038/s41586-025-09772-8 (PMC12823453; doi:10.1038/s41586-025-09772-8)
Supplement: Supplementary file 1 — This file contains Supplementary Figs. 1–11 (Uncropped immunoblots from main figures and extended data figures). [file 41586_2025_9772_MOESM1_ESM.pdf]

nature portfolio

https://doi.org/10.1038/s41586-025-09772-8

Supplementary information

ZAK activation at the collided ribosome

In the format provided by the authors and unedited

Huso et al., Supplementary Figure 1: Uncropped immunoblots from Figure 1

a

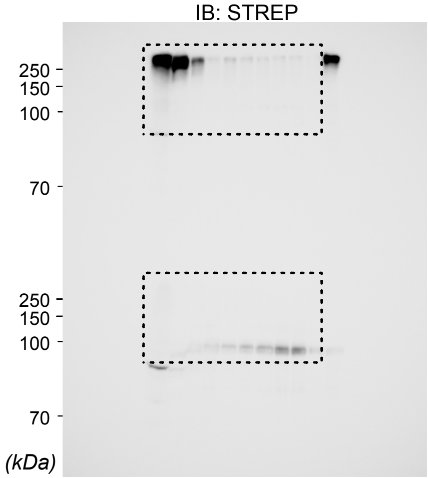

Supplementary Figure 1: Uncropped immunoblots from Figure 1
a, Immunoblot from Figure 1a of fractions collected from sucrose gradient sedimentation. Samples were HEK293T whole cell lysates transiently transfected with N-terminal STREP tagged WT (top) or T161A/S165A ZAK (bottom) expressed from full CMV promoter. Boxes indicate the areas shown in the final figures.

Huso et al., Supplementary Figure 2: : Uncropped immunoblots from Figure 2

a

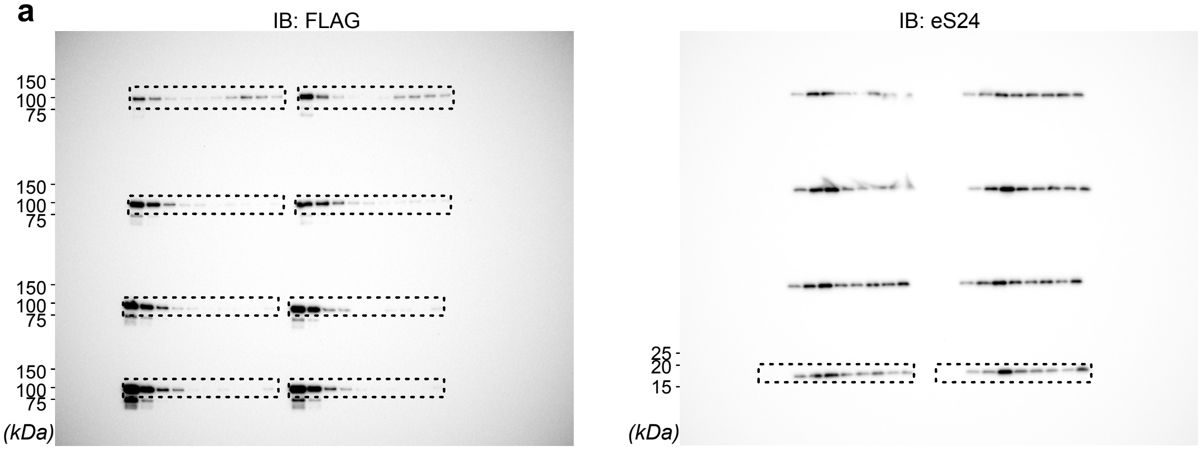

b

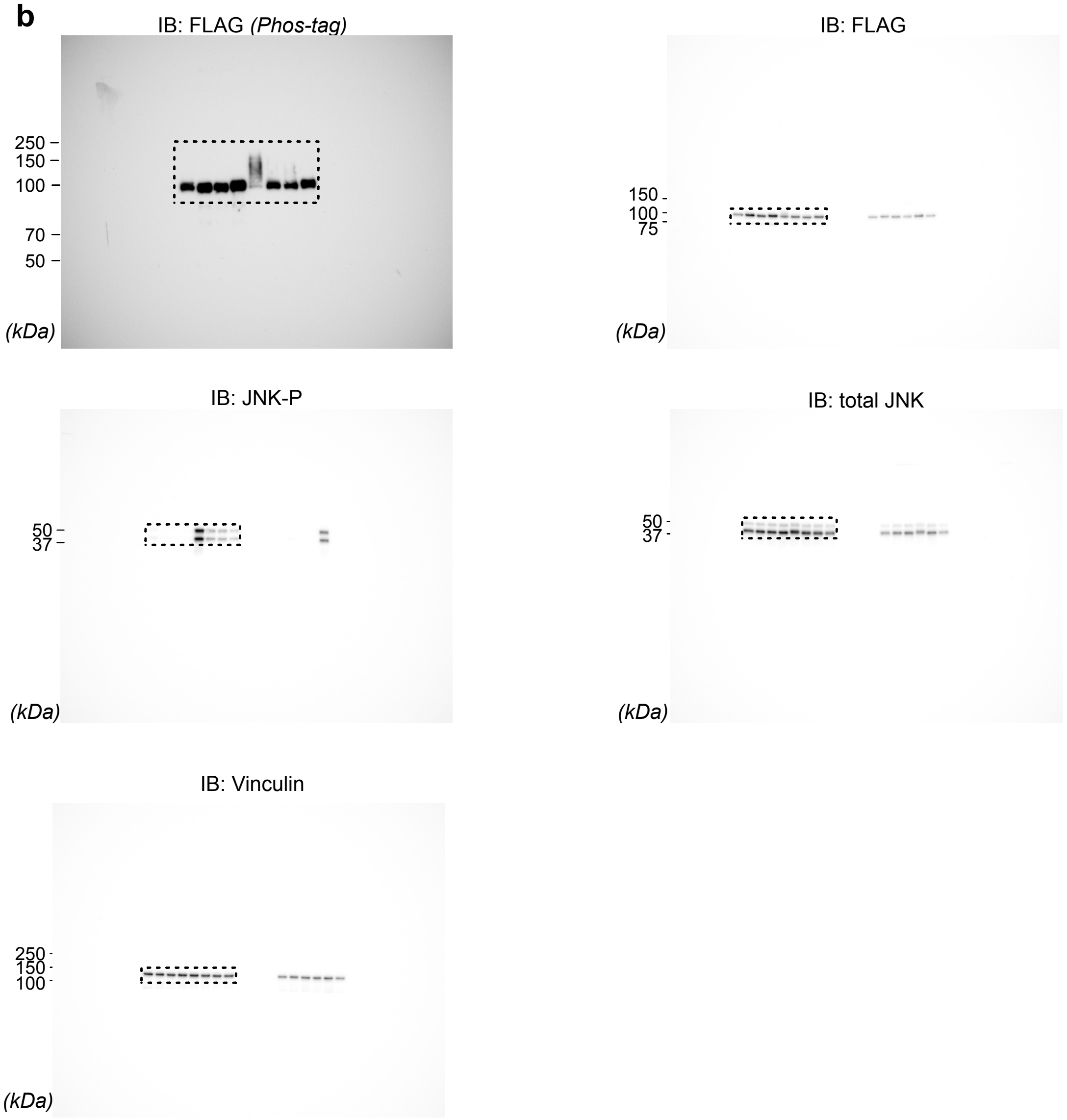

Supplementary Figure 2: Uncropped immunoblots from Figure 2

a, Immunoblots from Figure 2e of fractions collected from sucrose gradient sedimentation. Samples were from HEK293T ZAK KO whole cell lysates transiently transfected with N-terminal FLAG tagged (partial CMV promoter) WT (top), eS27-pin mutant, ES7-patch mutant, and pin+patch mutant (bottom). Left blot probed with FLAG antibody and right blots probed with eS24 antibody. Remaining eS24 blots are shown in Extended Data Figure 3b (Supplementary Figure 10a). **b,** Immunoblots from Figure 2f of whole cell lysates (same samples as Figure 2e). Boxes indicate the areas shown in the final figures.

Huso et al., Supplementary Figure 3: Uncropped immunoblots from Figure 3e and Figure 3f

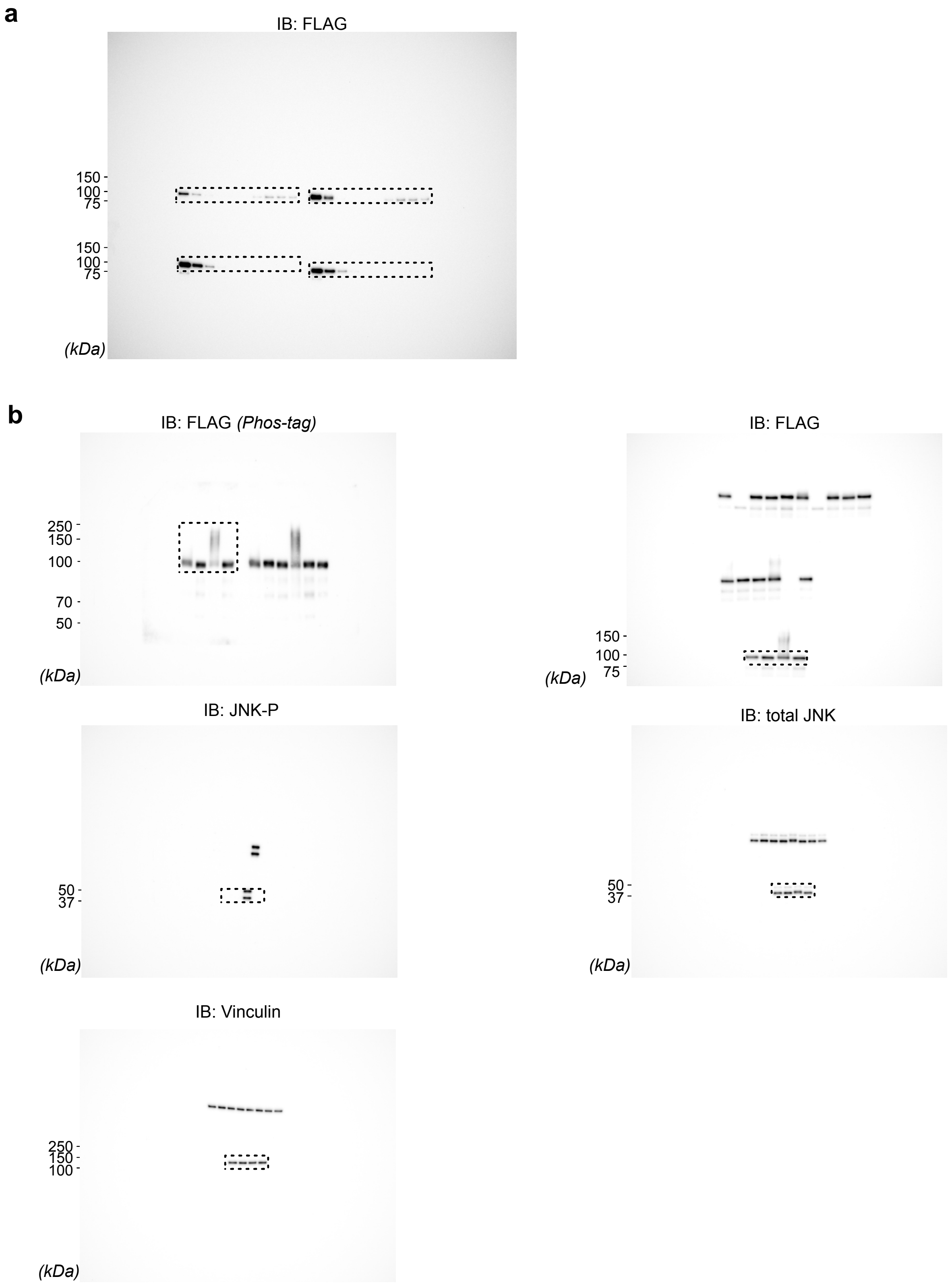

Supplementary Figure 3: Uncropped immunoblots from Figure 3e and Figure 3f
a, Immunoblots from Figure 3e of fractions collected from sucrose gradient sedimentation. Samples were from HEK293T ZAK KO whole cell lysates transiently transfected with N-terminal FLAG tagged (partial CMV promoter) WT (top two boxes) and RIH mutant (bottom two boxes). Membrane was probed with FLAG antibody. eS24 blots are shown in Extended Data Figure 3c (Supplementary Figure 10b). **b,** Immunoblots from Figure 3f of whole cell lysates (same samples as Figure 3e). Boxes indicate the areas shown in the final figures.

a

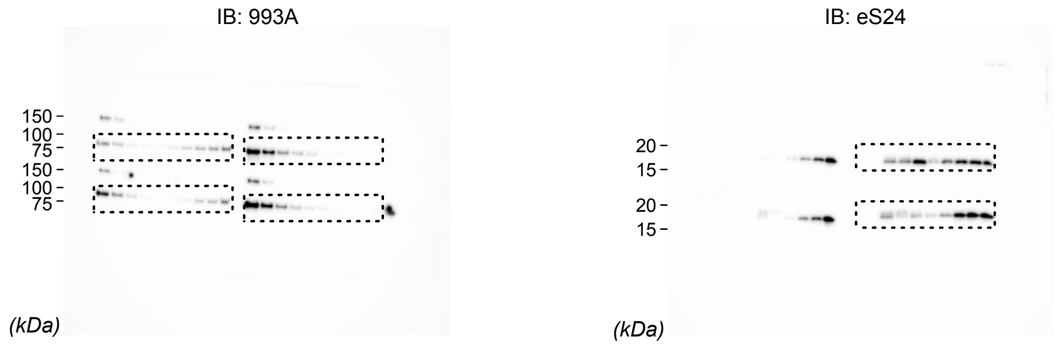

b

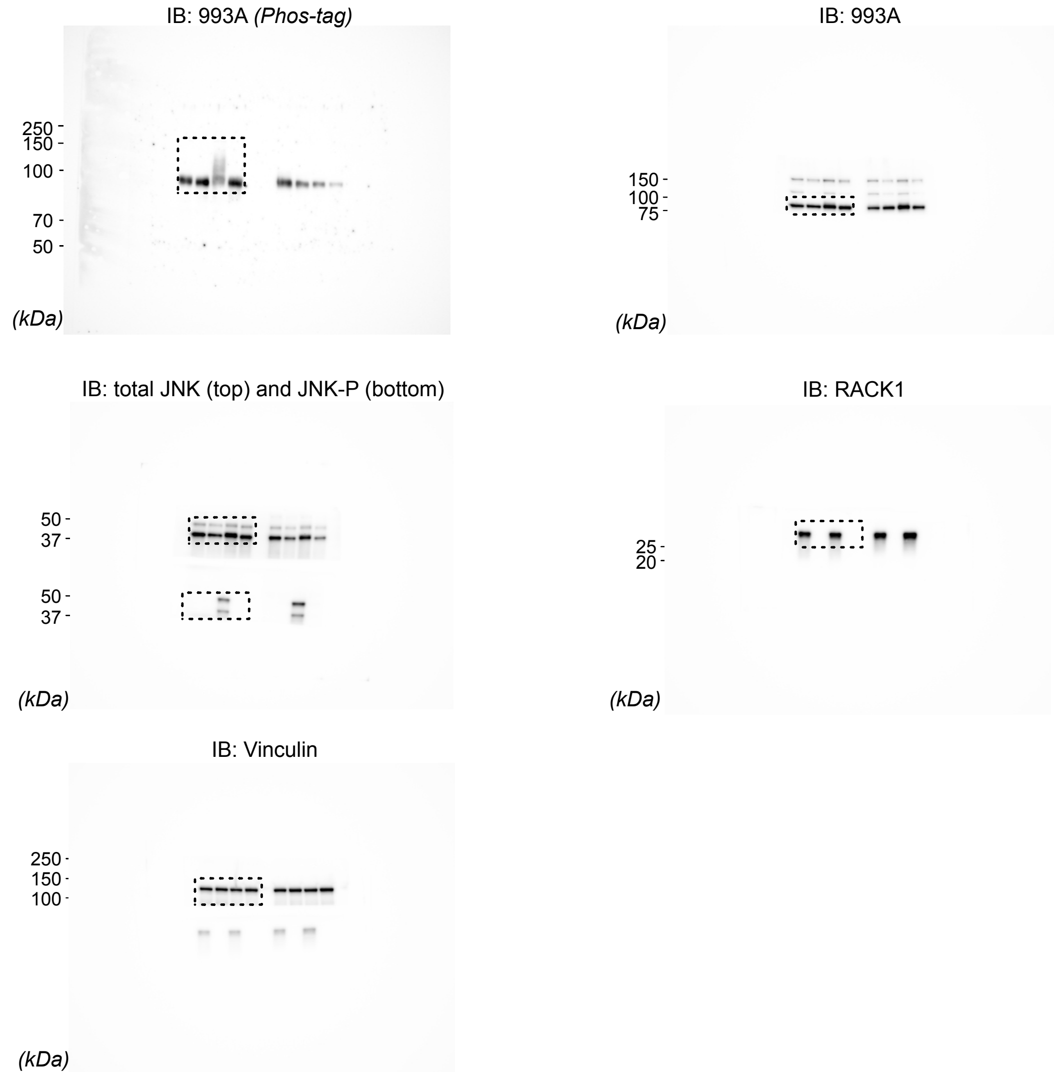

Supplementary Figure 4: Uncropped immunoblots from Figure 3g and Figure 3h
a, Immunoblots from Figure 3g of fractions collected from sucrose gradient sedimentation. Samples were from either WT HEK293T or RACK1 KO HEK293T whole cell lysates. 993A antibody was used (left blot) to probe for endogenous ZAK. Remaining eS24 blots are shown in Extended Data Figure 3d (Supplementary Figure 10c). **b,** Immunoblots from Figure 3h of whole cell lysates from either WT HEK293T or RACK1 KO HEK293T whole cell lysates. Boxes indicate the areas shown in the final figures.

Huso et al., Supplementary Figure 5: Uncropped immunoblots from Figure 4e and Figure 4f

a

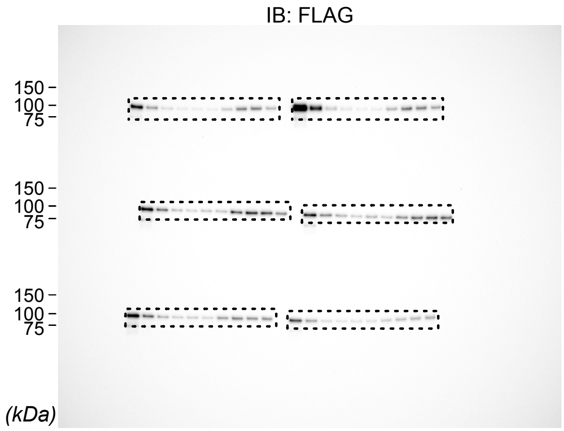

b

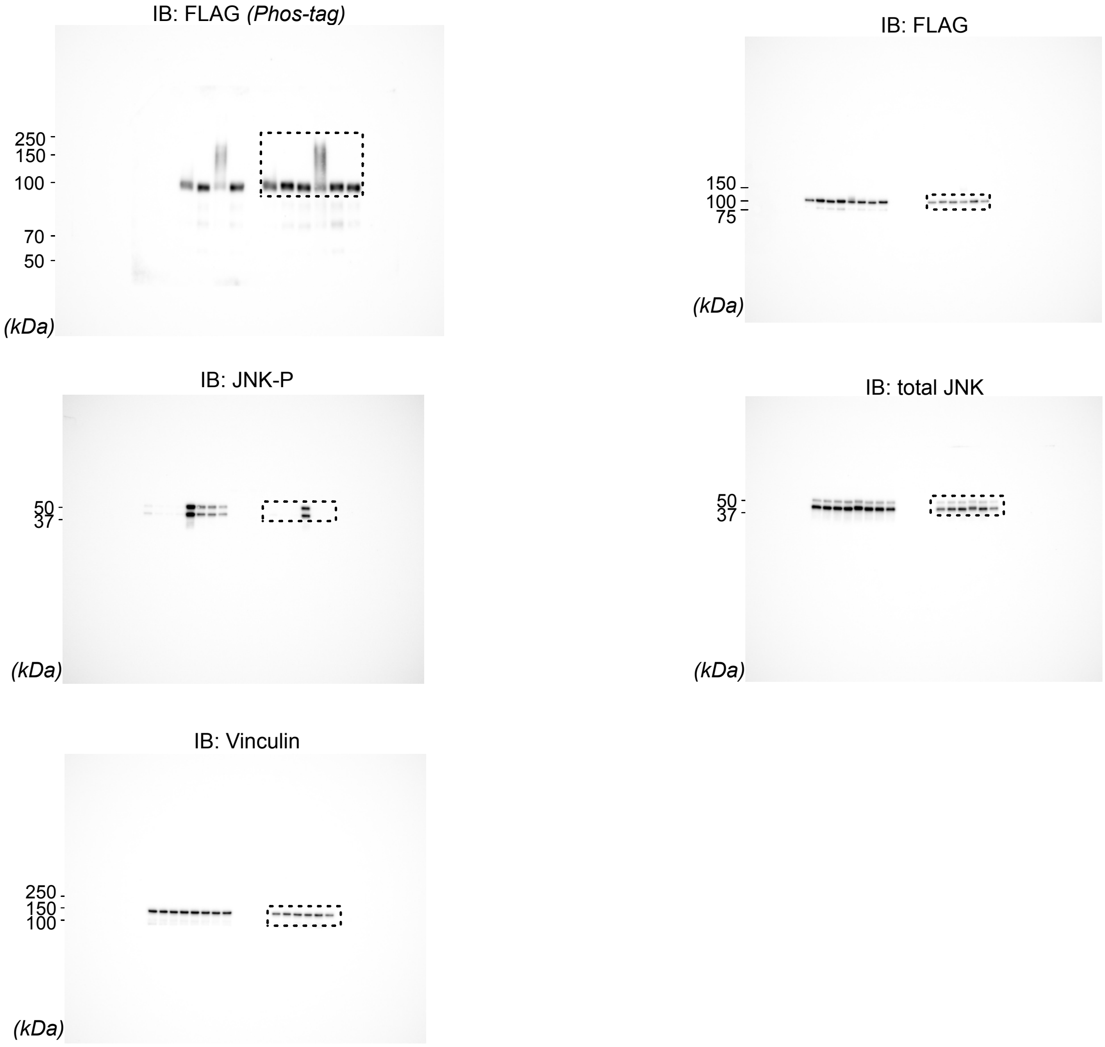

Supplementary Figure 5: Uncropped immunoblots from Figure 4e and Figure 4f
a, Immunoblots from Figure 4e of fractions collected from sucrose gradient sedimentation. Samples were from HEK293T ZAK KO whole cell lysates transiently transfected with N-terminal FLAG tagged (partial CMV promoter) WT (top two boxes), RIM mutant 1 (middle two boxes) and RIM mutant 2 (bottom two boxes). eS24 blots are shown in Extended Data Figure 3e (Supplementary Figure 10d). **b,** Immunoblots from Figure 4f of whole cell lysates (same samples as Figure 4e). Boxes indicate the areas shown in the final figures.

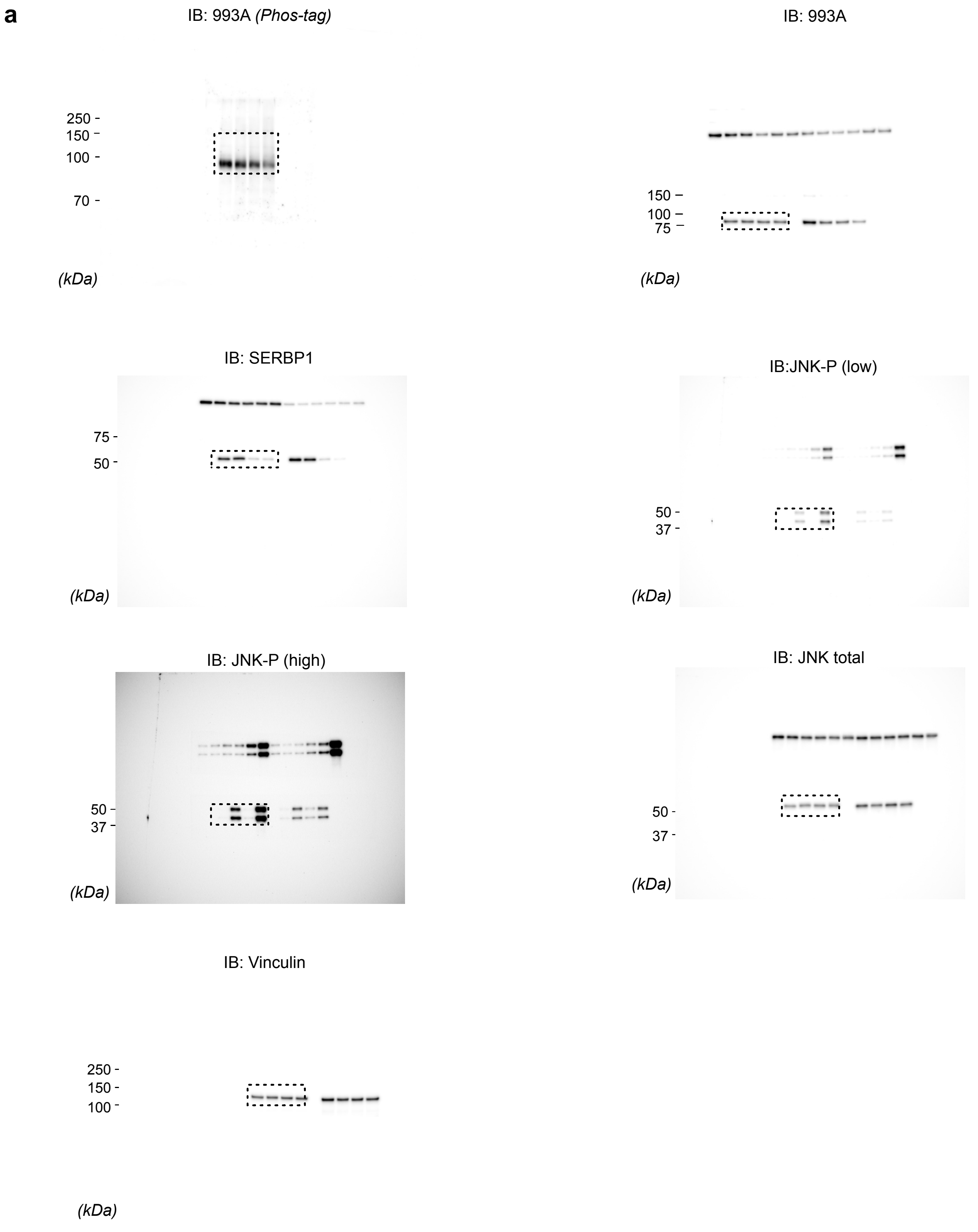

Supplementary Figure 6: Uncropped immunoblots from Figure 4i
a, Immunoblots from Figure 4i of whole cell lysates from HEK293T cells that were treated with either scramble siRNA or SERBP1 siRNA. Two different exposures of the anti-JNK-P blot were shown in Figure 4i and both exposures are include here. Boxes indicate the areas shown in the final figures.

a

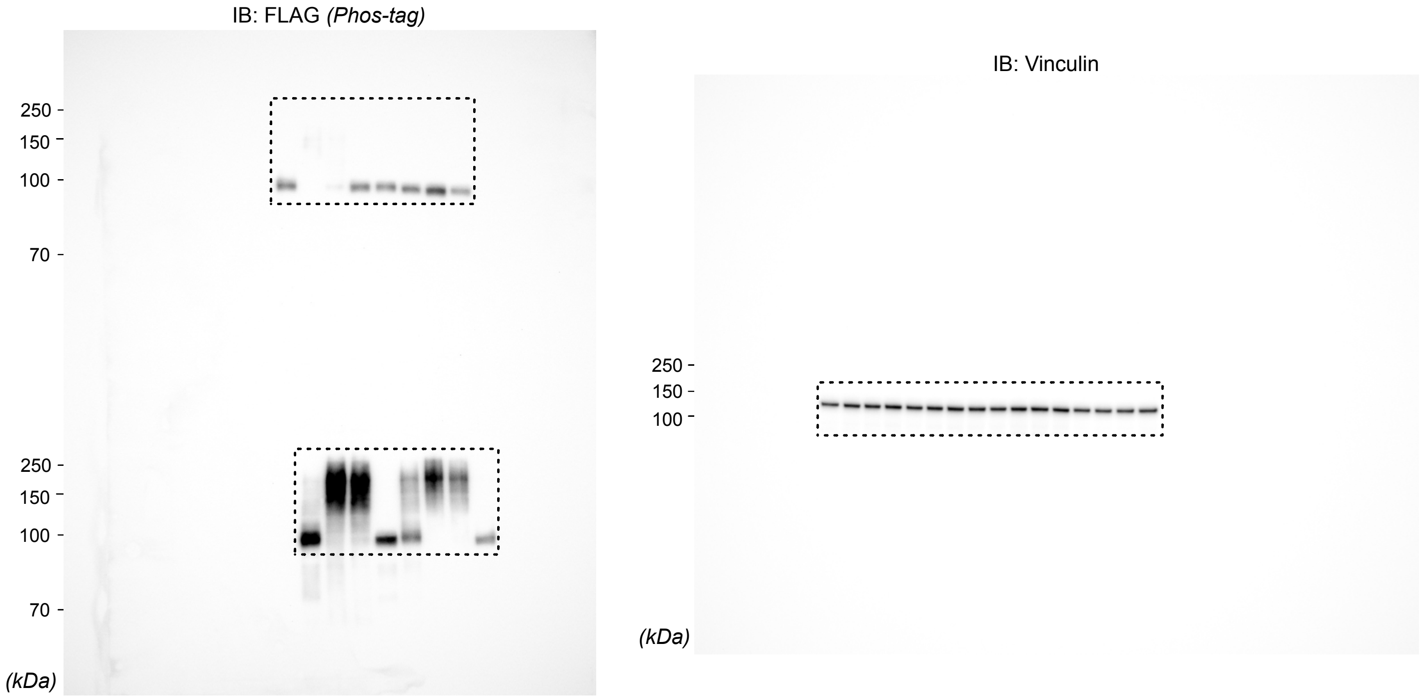

b

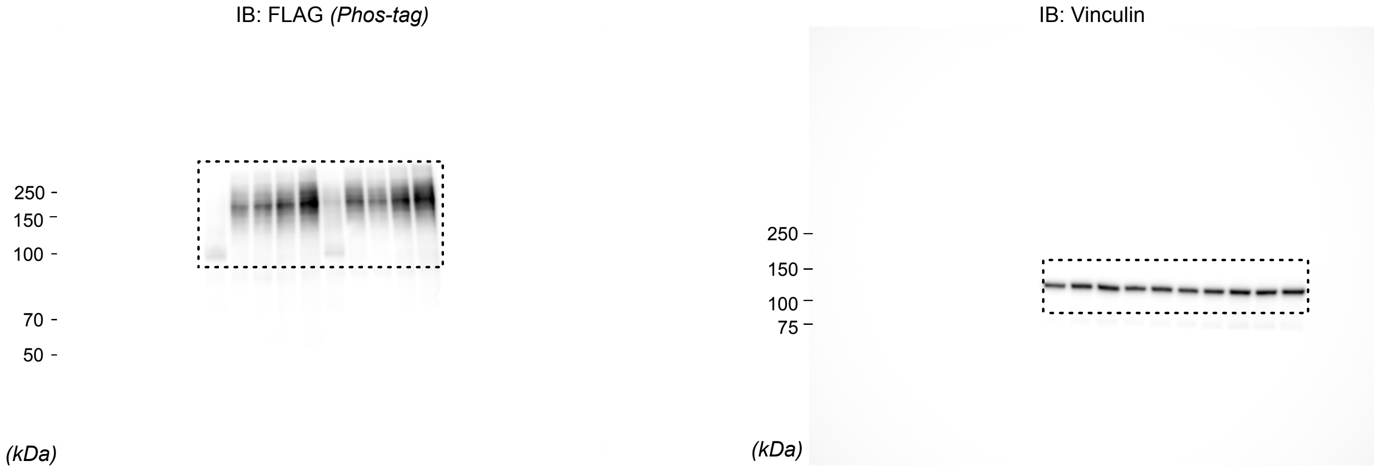

Supplementary Figure 7: Uncropped immunoblots from Figure 5d and Figure 5f
a, Immunoblots from Figure 5d of whole cell lysates from HEK293T ZAK KO cells transiently transfected with various FLAG ZAK constructs (partial CMV promoter). Samples for FLAG Phos-tag did not fit on one gel, so two gels were used and imaged at same time. **b,** Immunoblots from Figure 5f of whole cell lysates from HEK293T ZAK KO cells transiently transfected with various FLAG ZAK constructs (partial CMV promoter). Boxes indicate the areas shown in the final figures.

Huso et al., Supplementary Figure 8: Uncropped immunoblots/gels from Extended Data Fig 1a and Extended Data Fig 1b

a

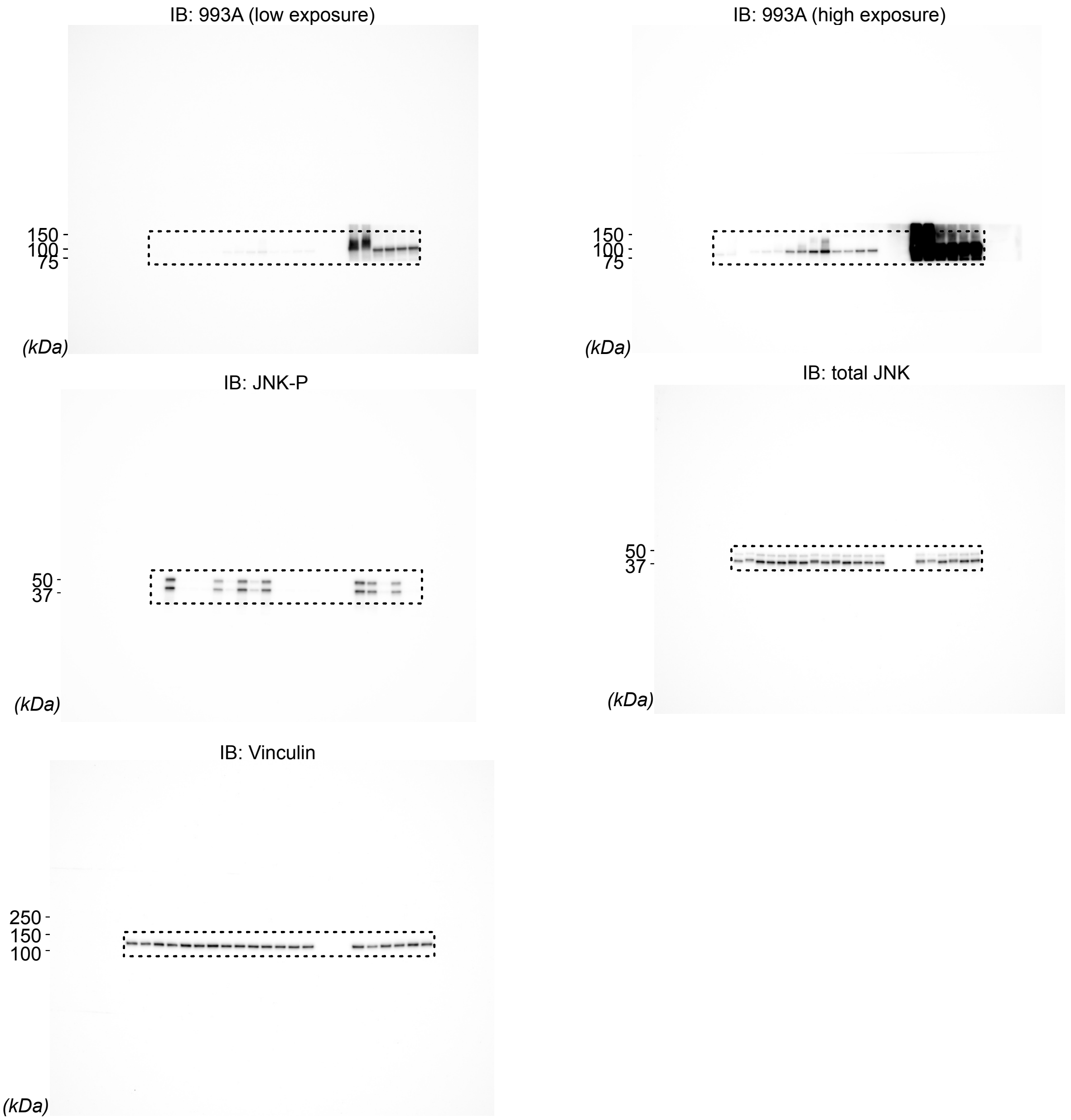

b

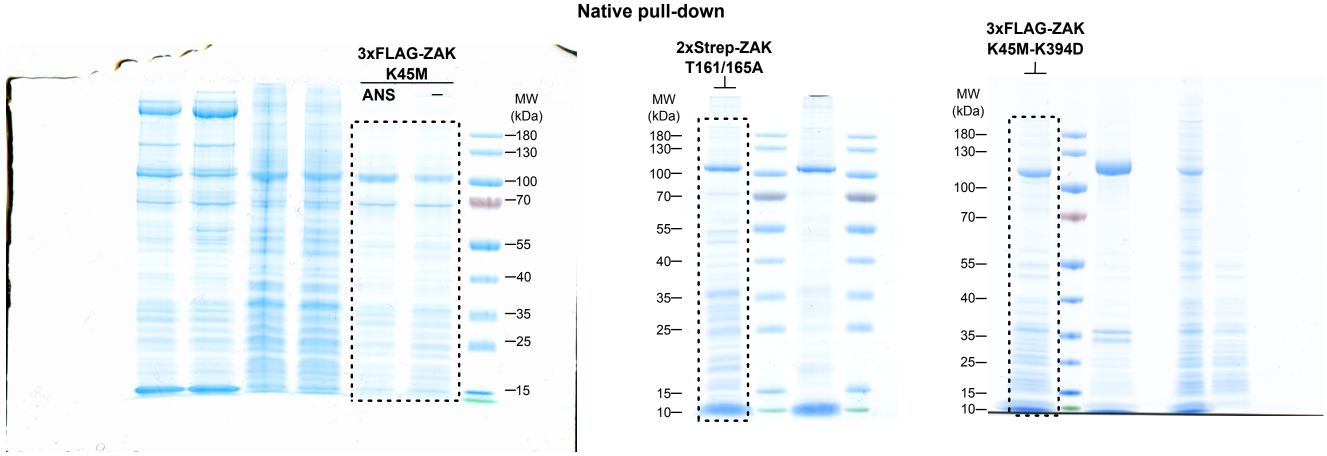

Supplementary Figure 8: Uncropped immunoblots/gels from Extended Data Fig 1a and Extended Data Fig 1b

a, Immunoblots from Extended Data Figure 1a of whole cell lysates from either WT HEK293T cells or ZAK KO HEK293T cells with various transient transfections and conditions. Two exposures were shown using the 993A antibody, so both blots are shown here. **b,** Coomassie-stained SDS-PAGE gels from Extended Data Figure 1b for native pull-down of tagged ZAK-K45M (left), ZAK-T161/165A (middle), ZAK-K45M-K394D (right). Boxes indicate the areas shown in the final figures.

Huso et al., Supplementary Figure 9: Uncropped immunoblots/gels from Extended Data Fig 1d and Extended Data Fig 1e and Extended Data Fig 1f

a

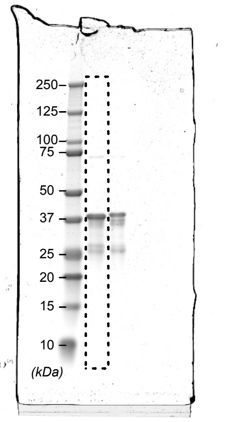

b

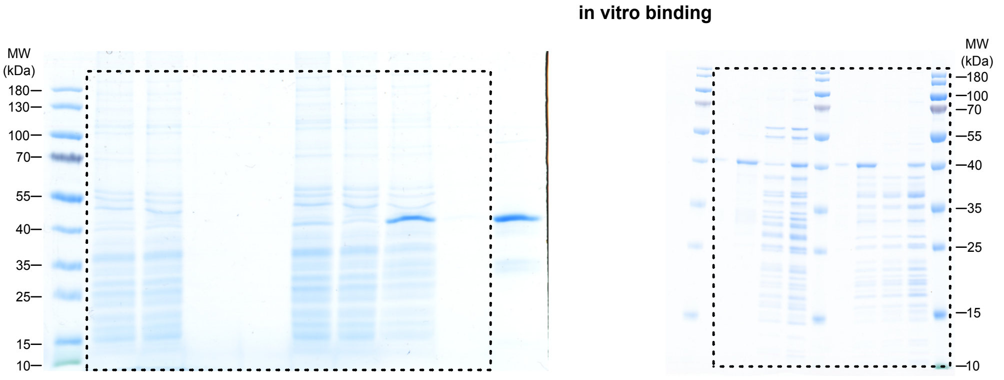

c

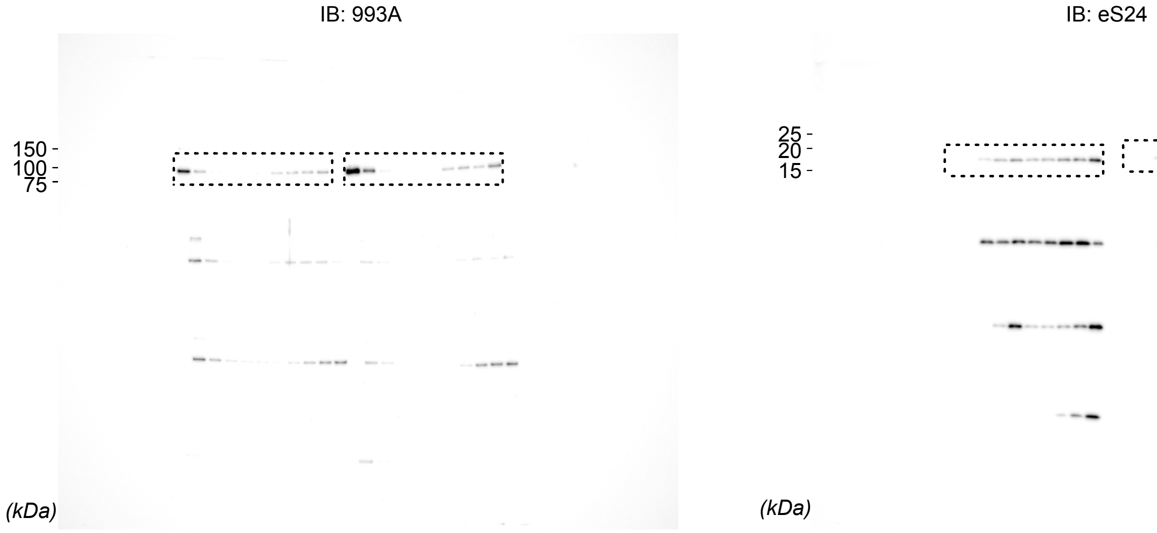

Supplementary Figure 9: Uncropped immunoblots/gels from Extended Data Fig 1d and Extended Data Fig 1e and Extended Data Fig 1f

a, Coomassie-stained SDS-PAGE gels from Extended Data Figure 1d for GST-RBR purification. **b,** Coomassie-stained SDS-PAGE gels from Extended Data Figure 1e for in vitro binding using purified 80S ribosomes (left) or ribosomal subunits (right) and purified GST-RBR. **c,** Immunoblots from Extended Data Figure 1f of fractions collected from sucrose gradient sedimentation. Samples were from whole cell lysates of WT HEK293T cells. Boxes indicate the areas shown in the final figures.

a

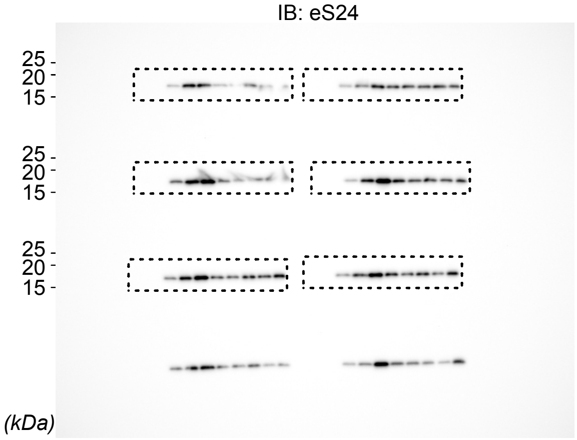

b

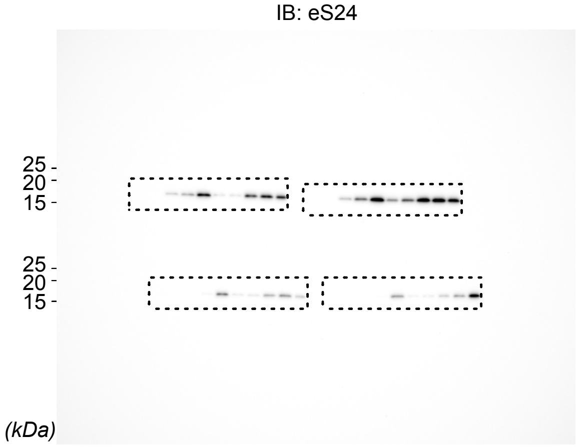

c

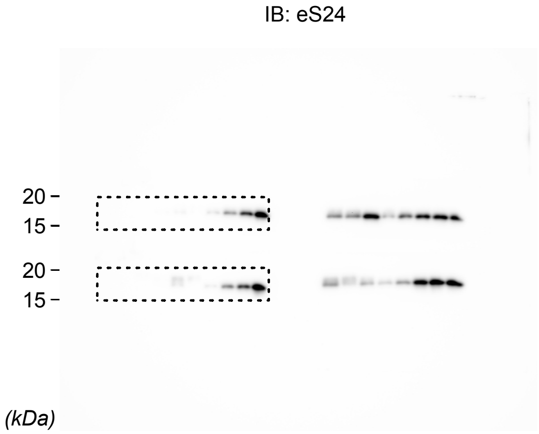

d

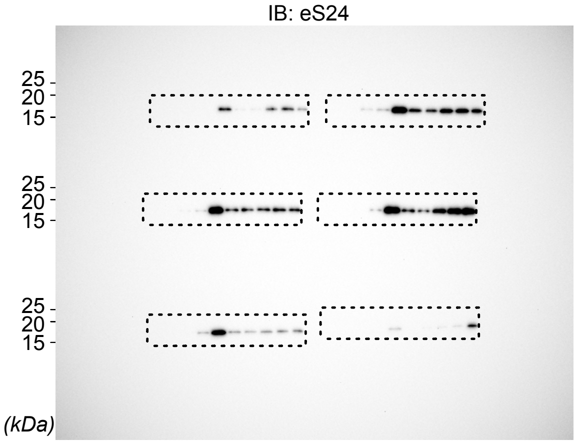

Supplementary Figure 10: Uncropped immunoblots from Extended Data Figure 3

a, Immunoblots from Extended Data Figure 3b of whole cell lysates (same samples as Figure 2e, 2f). **b,** Immunoblots from Extended Data Figure 3c of whole cell lysates (same samples as Figure 3e, 3f). **c,** Immunoblots from Extended Data 3d of whole cell lysates (same samples as Figure 3g, 3h). **d,** Immunoblots from Extended Data 3e of whole cell lysates (same samples as Figure 4e, 4f). Boxes indicate the areas shown in the final figures.

Huso et al., Supplementary Figure 11: Uncropped immunoblots from Extended Data Figure 7

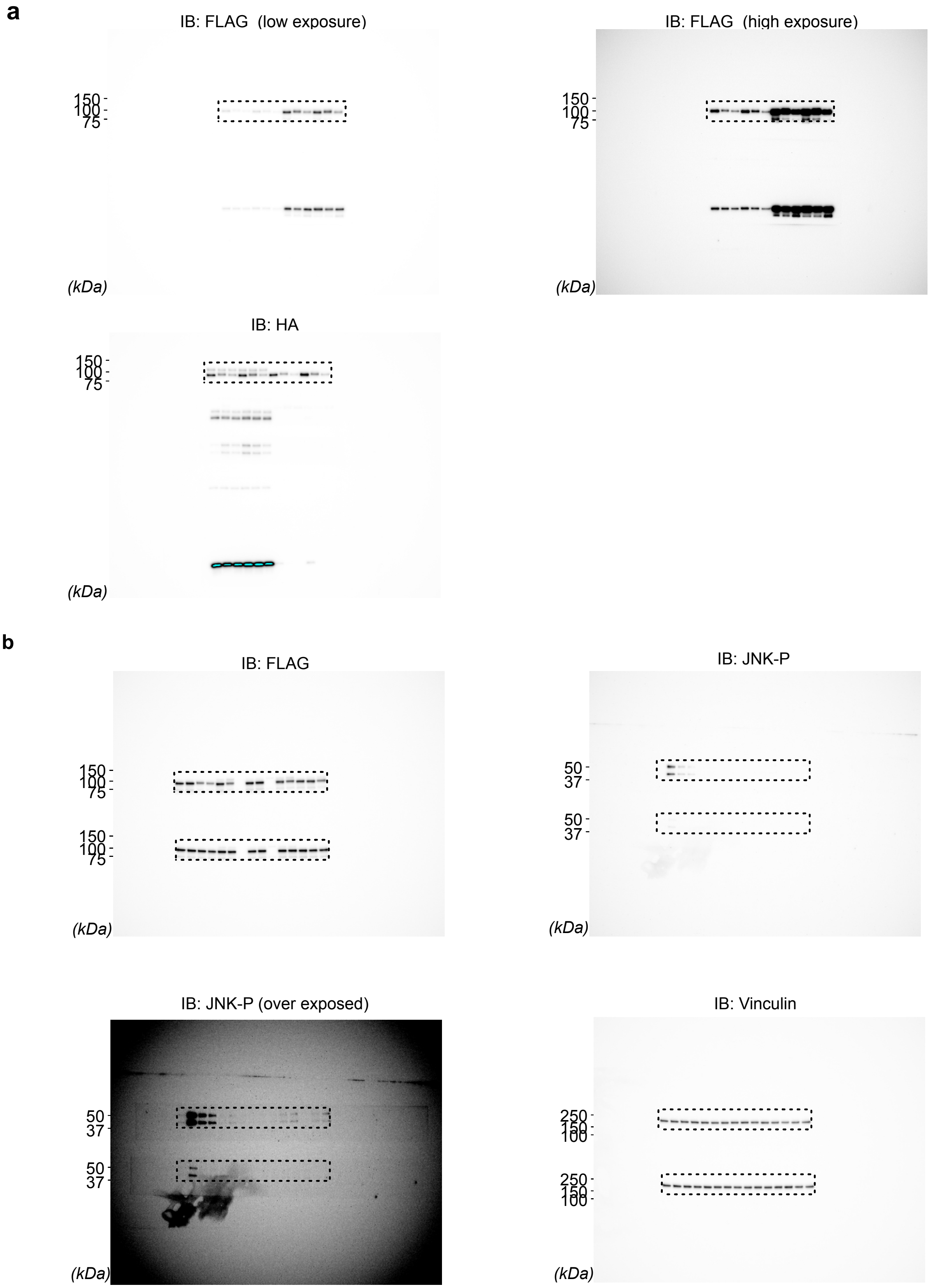

Supplementary Figure 11: Uncropped immunoblots from Extended Data Figure 7
a, Immunoblots from Extended Data Figure 7a of co-immunoprecipitation of FLAG tagged or HA tagged ZAK transiently transfected in HEK293T ZAK KO cells. **b,** Immunoblots from Extended Data Figure 7b of whole cell lysates transiently transfected with FLAG tagged ZAK constructs (partial CMV promoter). Boxes indicate the areas shown in the final figures.
