# Supplementary material for: ZAK activation at the collided ribosome
Source: Nature. 2025 Nov 19;649(8098):1051–60. doi: 10.1038/s41586-025-09772-8 (PMC12823453; doi:10.1038/s41586-025-09772-8)
Supplement: Supplementary file 2 — Reporting Summary [file 41586_2025_9772_MOESM2_ESM.pdf]

nature portfolio

Corresponding author(s): Dr. Roland Beckmann
Dr. Rachel Green

Last updated by author(s): Oct 1, 2025

Reporting Summary

Nature Portfolio wishes to improve the reproducibility of the work that we publish. This form provides structure for consistency and transparency in reporting. For further information on Nature Portfolio policies, see our Editorial Policies and the Editorial Policy Checklist.

Statistics

For all statistical analyses, confirm that the following items are present in the figure legend, table legend, main text, or Methods section.

n/a	Confirmed	
☐	☒	The exact sample size (*n*) for each experimental group/condition, given as a discrete number and unit of measurement
☐	☒	A statement on whether measurements were taken from distinct samples or whether the same sample was measured repeatedly
☐	☒	The statistical test(s) used AND whether they are one- or two-sided<br>*Only common tests should be described solely by name; describe more complex techniques in the Methods section.*
☒	☐	A description of all covariates tested
☐	☒	A description of any assumptions or corrections, such as tests of normality and adjustment for multiple comparisons
☐	☒	A full description of the statistical parameters including central tendency (e.g. means) or other basic estimates (e.g. regression coefficient) AND variation (e.g. standard deviation) or associated estimates of uncertainty (e.g. confidence intervals)
☒	☐	For null hypothesis testing, the test statistic (e.g. F, t, r) with confidence intervals, effect sizes, degrees of freedom and P value noted<br>*Give P values as exact values whenever suitable.*
☒	☐	For Bayesian analysis, information on the choice of priors and Markov chain Monte Carlo settings
☒	☐	For hierarchical and complex designs, identification of the appropriate level for tests and full reporting of outcomes
☒	☐	Estimates of effect sizes (e.g. Cohen's *d*, Pearson's *r*), indicating how they were calculated

Our web collection on statistics for biologists contains articles on many of the points above.

Software and code

Policy information about availability of computer code

Data collection	Cryo-EM data were collected with EPU v.3.7 software.
Data analysis	Western blots and coomassie gels were processed using: Image J 2.3. CLIP-seq data was processed using: mi_tools version 1.1.6; trim_galore version 0.6.10; STAR version 2.7.3a; samtools version 1.9; R version 4.4.1. Cryo-EM data was processed using: MotionCor2 (version 1.4.0), CTFFIND4 (version 4.1.13), RELION (version 5.0 beta), CryoSPARC (version 4.6.0).<br>Molecular models were built and refined using WinCoot 0.9.8.93 and Phenix1.20.1-4487.<br>AlphaFold database (https://alphafold.ebi.ac.uk/), AlphaFold3 and AlphaFold2 Multimer were used for initial model prediction and its multimer implementation to analyze protein-protein interactions. Structural figures were created using Chimera X v1.9.

For manuscripts utilizing custom algorithms or software that are central to the research but not yet described in published literature, software must be made available to editors and reviewers. We strongly encourage code deposition in a community repository (e.g. GitHub). See the Nature Portfolio guidelines for submitting code & software for further information.

Data

Policy information about availability of data

All manuscripts must include a data availability statement. This statement should provide the following information, where applicable:

- Accession codes, unique identifiers, or web links for publicly available datasets
- A description of any restrictions on data availability
- For clinical datasets or third party data, please ensure that the statement adheres to our policy

The CLIP-seq data generated in this study have been deposited in the Gene Expression Omnibus (GSE299329). The cryo-EM structural data generated in this study have been deposited in the Electron Microscopy Data Bank and the Protein Data Bank (PDB), respectively, under the following accession codes: EMD-54172 for the composite ZAK-disome (obtained from kinase inactive ZAK pull-down with ANS treatment, PDB accession 9RPV); EMD-54140 for the stalled 80S and EMD-54141 for the collided 80S (related to composite map); EMD-54148 for the locally refined ZAK-RACK1 region of the stalled 80S and EMD-54147 for the locally refined ZAK-RACK1 region of the collided 80S (both related to composite map); EMD-ZZZZZ for
the EMD-54149 for the hybrid state translating 80S and EMD-54150 for the hibernating 80S (obtained from native FLAG-ZAKα-K45M pull-down); EMD-54236 for the locally refined hybrid state translating 80S (PDB accession 9RSX); EMD-54165 for the in vitro reconstituted ZAK-RBR-40S complex; EMD-54166 for the stalled 80S and EMD-54167 for the collided 80S (obtained from FLAG-ZAKα-K45M K394D pull-down with ANS treatment). The structures used for atomic model building of ZAK-bound disome complexes are available from Worldwide Protein Data Bank (wwPDB) with accession codes 6Y57, 7QVP, 8GLP. All other data are presented in the main text of the manuscript (Figures 1-5) as well as supplemental (Extended Figures 1-9 and Supplemental Table 1 and Supplementary Information Documents).

Research involving human participants, their data, or biological material

Policy information about studies with human participants or human data. See also policy information about sex, gender (identity/presentation), and sexual orientation and race, ethnicity and racism.

Reporting on sex and gender	n/a
Reporting on race, ethnicity, or other socially relevant groupings	n/a
Population characteristics	n/a
Recruitment	n/a
Ethics oversight	n/a

Note that full information on the approval of the study protocol must also be provided in the manuscript.

Field-specific reporting

Please select the one below that is the best fit for your research. If you are not sure, read the appropriate sections before making your selection.

☒ Life sciences ☐ Behavioural & social sciences ☐ Ecological, evolutionary & environmental sciences

For a reference copy of the document with all sections, see nature.com/documents/nr-reporting-summary-flat.pdf

Life sciences study design

All studies must disclose on these points even when the disclosure is negative.

Sample size	For biochemistry and cell-based assays, no statistical methods or calculations were utilyzed. All experimental results were successfully replicated at least twice. The number of biological replicates was a minimum of two per assay. See "Statistics and Reproducibility" section for complete information. For Cryo-EM datasets sufficient number of micrographs were collected to achieve the reported resolution of map. Complete Cryo-EM statistics are provided in Extended Data Table 1.
Data exclusions	During cryo-EM data processing, particles were excluded if 2D class averages represented noise or did not show clearly identifiable features for 80S particles.
Replication	See Methods and the section titled "Statistics and Reproducibility" for details on replications. As discussed, all experiments were replicated on different days (biological replicates) in the same or similar experiments. All cryo-EM structures were determined from independent half datasets, which were compared to assess the resolution of the reconstruction.
Randomization	Randomization does not apply for biochemistry and cell-based assays in this project. To determine the overall resolution for cryo-EM reconstructions the "Gold standard" Fourier shell correlation (FSC) was used with a 0.143 cutoff criterion. Here, particle sets are randomly divided to generate two independent 3D maps that were used to calculate the FSC.
Blinding	No blinding was required for the reported experiments and therefore not attempted.

Reporting for specific materials, systems and methods

We require information from authors about some types of materials, experimental systems and methods used in many studies. Here, indicate whether each material, system or method listed is relevant to your study. If you are not sure if a list item applies to your research, read the appropriate section before selecting a response.

Materials & experimental systems

n/a	Involved in the study
☐	☒ Antibodies
☐	☒ Eukaryotic cell lines
☒	☐ Palaeontology and archaeology
☒	☐ Animals and other organisms
☒	☐ Clinical data
☒	☐ Dual use research of concern
☒	☐ Plants

Methods

n/a	Involved in the study
☒	☐ ChIP-seq
☒	☐ Flow cytometry
☒	☐ MRI-based neuroimaging

Antibodies

Antibodies used

Primary antibodies used:
Rabbit anti-eS24 (Abcam, Cat# ab196652, 1:1000)
Mouse anti-FLAG (Sigma, Cat#A8592, 1:5000)
Rabbit anti-HA (Cell Signaling, Cat#3724, 1:1000)
Mouse anti-JNK1 (Cell Signaling, Cat #3708, 1:1000)
Rabbit anti-Phospho-SAPK/JNK (Cell Signaling, Cat# 4668S, 1:1000)
Rabbit anti-RACK1 (Cell Signaling, Cat# 5432S, 1:1000)
Rabbit anti-SERBP1 (Novus, Cat# NBP1-85660, 1:1000)
Mouse anti-STREP (Sigma, Cat# 71591-3, 1:5000)
Mouse anti-Vinculin (Santa Cruz, Cat# sc-73614, 1:2000)
Rabbit anti-ZAK (Fortis, Cat# A301-993A, 1:1000)

Secondary antibodies used:
anti-Mouse (Cell Signaling, Cat# 7076S, 1:5000)
anti-Rabbit (Cell Signaling, Cat# 7074S, 1:5000)

Validation

anti-eS24 - Validated for IB by manufacturer, by publications listed on manufacturer website
anti-FLAG - Validated for IB by manufacturer, by experiments in Green lab (unpublished)
anti-HA - Validated for IB by manufacturer using SimpleChIP® Enzymatic Chromatin IP Kits
anti-JNK1 - Validated for IB by manufacturer, by publications listed on manufacturer website
anti-Phospho-SAPK/JNK - Validated for IB by manufacturer, by publications listed on manufacturer website
anti-RACK1 - Validated for IB by manufacturer, and by knockout experiments in this manuscript
anti-SERBP1 - Validated for IB by manufacturer and by siRNA knockdown experiment in this manuscript
anti-STREP - Validated for IB by manufacturer, by publications listed on manufacturer website
anti-Vinculin - Validated for IB by manufacturer, by publications listed on manufacturer website
anti-ZAK - Validated for IB by manufacturer and by knockout experiments in this manuscript (and unpublished)
anti-Mouse - Validated for IB by manufacturer using CST primary antibodies
anti-Rabbit - Validated for IB by manufacturer using CST primary antibodies

Eukaryotic cell lines

Policy information about cell lines and Sex and Gender in Research

Cell line source(s)	HEK293T cells (ATCC CRL-3216); EXPi293F cells (Thermo Fisher; A14527)
Authentication	ZAK knockout and RACK1 knockout were authenticated by sequencing and western blot.
Mycoplasma contamination	Cell lines were negative for mycoplasma
Commonly misidentified lines (See ICLAC register)	n/a

Plants

Seed stocks	n/a
Novel plant genotypes	n/a
Authentication	n/a
